# Rotary Friction Welding of Dissimilar Polymer Rods Containing Metal Powder

**DOI:** 10.3390/polym15224354

**Published:** 2023-11-08

**Authors:** Chil-Chyuan Kuo, Hong-Wei Chen, Song-Hua Huang

**Affiliations:** 1 Department of Mechanical Engineering, Ming Chi University of Technology, No. 84, Gungjuan Road, Taishan District, New Taipei City 24301, Taiwan; m10119022@mail.mcut.edu.tw; 2 Research Center for Intelligent Medical Devices, Ming Chi University of Technology, No. 84, Gungjuan Road, Taishan District, New Taipei City 24301, Taiwan; 3 Department of Mechanical Engineering, Chang Gung University, No. 259, Wenhua 1st Rd., Guishan Dist., Taoyuan City 33302, Taiwan; 4 Center for Reliability Engineering, Ming Chi University of Technology, No. 84, Gungjuan Road, Taishan District, New Taipei City 24301, Taiwan; 5 Li-Yin Technology Co., Ltd., No. 37, Lane 151, Section 1, Zhongxing Road, Wugu District, New Taipei City 24101, Taiwan

**Keywords:** rotary friction welding, polylatic acid, copper powder, aluminum powder, ambient temperature

## Abstract

Three-dimensional printing is widely used for manufacturing a variety of functional components. However, the 3D printing machine substantially limits the size of the functional components. Rotary friction welding (RFW) is a possible solution to this problem. In addition, there is a notable scarcity of research directed toward the domain knowledge of RFW involving dissimilar polymer rods containing metal powder. In this study, two welding specimens fabricated by polylactic acid (PLA)-containing copper powder and PLA-containing aluminum powder were joined using a turning machine. After RFW, a bending test and a Shore A surface hardness test were performed to investigate the weld quality. It was found that the bending strength of the welded parts fabricated by RFW of PLA and PLA-containing Al powder rods can be enhanced by about 57.5% when the welded part is placed at 45 °C. Surface hardness test results showed that the surface hardness of the weld interface is better than that of the 3D printed parts, and the average surface hardness of the weld interface from RFW of PLA and PLA is the highest. The surface hardness of the weld joint is about 3% higher than that of the base material. The surface hardness of the heat-affected zone is about 3% lower than that of the base material. The average peak temperature of the welded joint is the highest in the RFW of PLA-containing Al powder and PLA-containing Al powder rods. The average peak temperature of the weld joint can be as high as 160 °C. The average peak temperature of the welded joint is the highest in the RFW of PLA-containing Cu powder and PLA-containing Cu powder rods. The average peak temperature of the welded joint can be as high as 144 °C. A technical database was built for the selection of ambient temperatures used for the RFW of dissimilar polymer rods containing metal powder and three base materials.

## 1. Introduction

Welding is a fabrication process that is widely used on thermoplastics or metals. Rotary friction welding (RFW) [1] is a green welding process compared with arc welding [2], which is widely used in many industries, such as the aircraft industry and the electrical industry [3]. It is well known that RFW is used to fabricate truck roller bushes, mining tools, and automotive tubes, shafts, and piston rods [4,5]. RFW minimizes heat and friction exposure on the components during welding, thereby reducing grain formation [6]. The proposed method can be used to join plastics to metals using a machined metal interface [7]. Skowronska et al. [8] investigated the structural properties of welded joints. The results showed that the surface hardness in a welded joint exceeding HV 340 was achieved using high-speed friction welding. Eliseev and colleagues [9] investigated the microstructural changes within the transfer layer of aluminum alloy welds. Their findings revealed a decrease in the grain size of incoherent intermetallic particles and a reduction in volume fraction towards the layer’s center. Anwar et al. [10] examined the microstructure of post-weld heat-treated rotary friction welds in alloy 800H. The outcomes demonstrated the successful attainment of the minimum grain size through post-weld heat treatment. Khalaf et al. [11] examined the heat generation in various tool parts. The findings revealed that pins with more edges, particularly triangular ones, exhibited greater heat generation compared to pins with smoother shapes. This heightened heat generation resulted in increased heat flux on the surface of the high-density polyethylene. Vidakis et al. [12] explored the effects of welding tool pin geometry, travel speed, and rotational speed on acrylonitrile butadiene styrene, which was fabricated using the material extrusion process [13]. Ma et al. [14] investigated the impact of temperature on the mechanical properties of aluminum alloy joints produced through friction stir welding. The findings indicated that a decrease in temperature gradient along the thickness, attributed to the presence of the pinhole, led to increased heat input and material flow at the lower section. Moreover, the results demonstrated that the welded specimens exhibited enhanced mechanical strength when compared to non-welded three-dimensional printed specimens of similar geometry. Yang et al. [15] conducted an analysis of the contact behavior and temperature characteristics in welding through the application of the harmonic balance method. Both simulation and experimental outcomes emphasized the significance of welding time and amplitude as pivotal factors influencing interface temperature. It was observed that increasing both amplitude and welding time led to a substantial rise in the interface temperature. Maggiore et al. [16] conducted a review focusing on structural adhesive joints within hybrid joining processes. Their review highlights that the adoption of hybrid joining technology offers promising potential as a solution across various industries for the reduction of manufacturing costs. Pereira et al. [17] conducted an analysis to explore how different welding parameters affect the morphology and mechanical strength of friction stir welds in polymers. Their findings indicated that an increased rotational speed-to-welding speed ratio contributed to enhanced joint efficiency. However, establishing precise mathematical relationships proved challenging due to the variability in welding conditions. Iftikhar et al. [18] categorized the existing literature on friction stir spot welding and friction stir welding of thermoplastic polymers and polymer composites based on tooling conditions, materials being joined, joint configurations, and environmental conditions. However, there is a notable scarcity of research directed toward the domain knowledge of RFW involving dissimilar polymer rods containing metal powder.

Polylactic acid (PLA) [19] is a biodegradable and bioactive thermoplastic made from renewable resources such as corn starch or sugarcane. It is a type of polyester and belongs to the family of polymers. PLA is frequently employed in various applications, including food packaging, medical implants, and as a biodegradable alternative to traditional plastics. In practice, PLA is the best choice for additive manufacturing using a material extrusion (MEX) [20], since it requires less energy for conversion than many petrochemical plastics. Thus, PLA plastic is suitable for making consumer products that need to withstand heavy use [21]. Generally, the reliability and validity [22] of the frictionally welded components are significantly related to the ambient temperatures [23,24]. Additive manufacturing is widely used for manufacturing a variety of functional components. MEX is a promising three-dimensional (3D) printing approach in industrial applications. However, the MEX substantially limits the size of the printed physical model. Adhesive bonding, mechanical fitting, large-area 3D printing, or welding are possible solutions. In practice, the welding technique provides benefits, such as building functional components with dissimilar materials and sustainable mechanical joints. Rotary friction welding (RFW) is regarded as an environmentally friendly and sustainable manufacturing technology in contrast to arc welding, as it offers reduced energy consumption and minimizes environmental pollution. Thus, investigating the welding properties of dissimilar polymer rods containing metal powder is an important research topic. Therefore, the primary goal of this study is to develop a comprehensive understanding of the RFW process for dissimilar polymer rods with incorporated metal powder. Two welding specimens fabricated by PLA-containing Al powder and PLA-containing Cu powder were joined using a conventional lathe. After RFW, Shore A surface hardness tests and bending tests were used to investigate the mechanical properties of the frictionally welded parts. Finally, a technical database was created to facilitate the selection of ambient temperatures for RFW of polymer rods containing different metal powders.

## 2. Experiment

**Figure 1:**
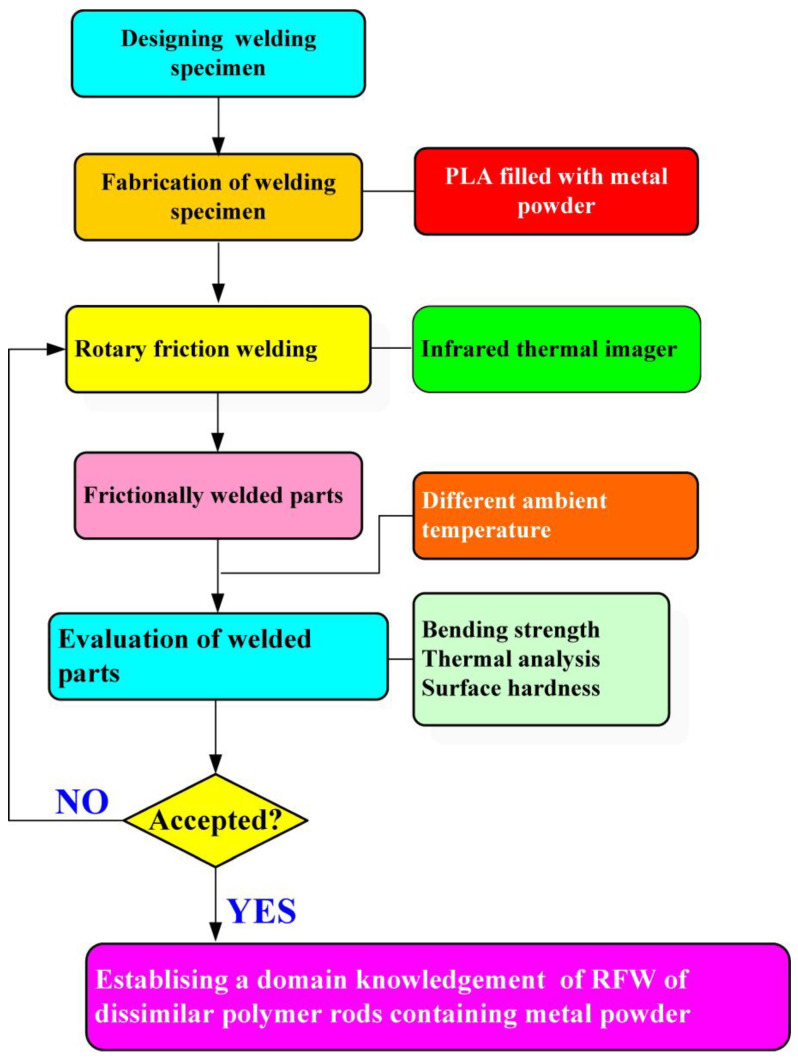
Flow diagram of the experimental process.

**Figure 2:**
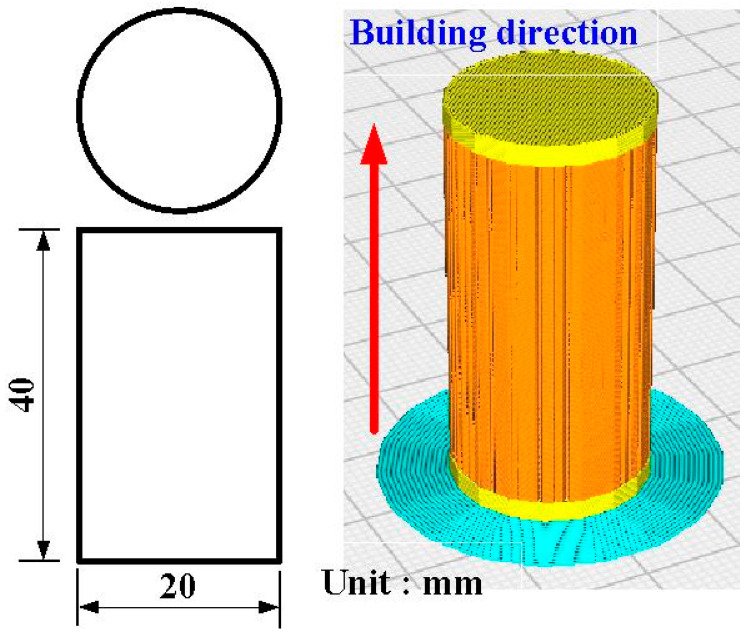
Three-dimensional digital model sliced by three-dimensional printing software.

**Figure 3:**
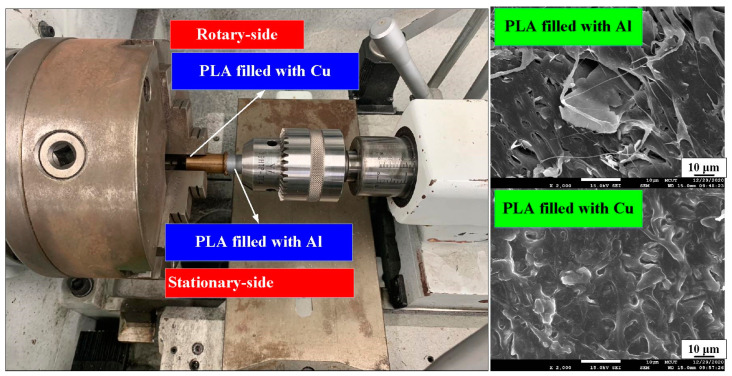
FRW of PLA-containing Al and PLA-containing Cu powders.

**Figure 4:**
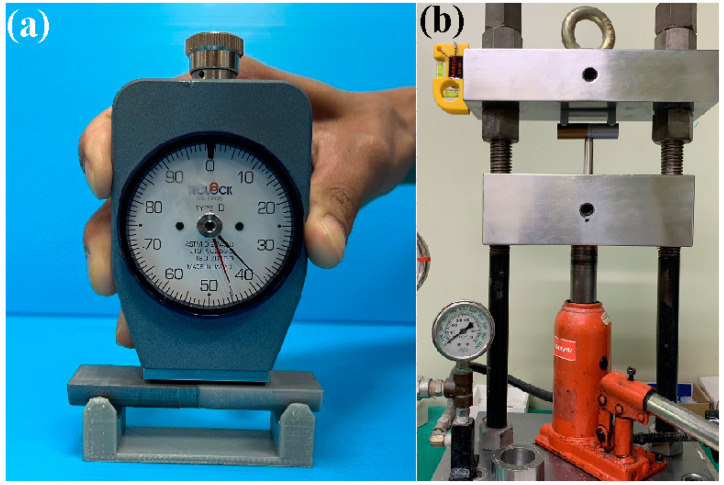
Experimental setup for (**a**) surface hardness and (**b**) bending strength tests of frictionally welded parts.

**Figure 5:**
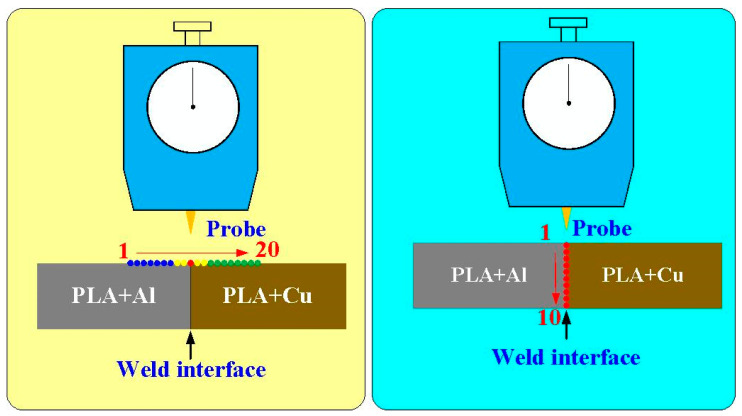
Schematic diagram of the surface hardness measurement location.

Figure 1 shows the flow diagram of the experimental process. Research items involve designing welding specimens, the fabrication of welding specimens, RFW, and evaluation of the welded parts. Finally, a comprehensive database of the RFW of dissimilar polymer rods containing metal powder has been successfully established. Two different kinds of polymer filament, i.e., PLA-containing Al powder and Cu powders (Thunder 3D Inc., New Taipei City, Taiwan), were used to print welding specimens in a three-dimensional printing apparatus [25]. These two materials are commercially available. The diameter of the filament is 1.75 mm. The length and diameter of the welding specimen is about 40 mm and 20 mm, respectively. Figure 2 shows that the 3D digital model was sliced by 3D printing software (Ultimaker Inc., Utrecht, The Netherlands). Based on many years of printing experience, the nozzle diameter of the MEX machine is 0.4 mm. The MEX process parameters for print welding samples using PLA-containing Al powder filament involve a printing bed temperature of 100 °C, printing speed of 80 mm/s, and printing temperature of 200 °C. The MEX process parameters for print welding samples using PLA-containing Cu powder filament involve a printing bed temperature of 100 °C, printing speed of 80 mm/s, and printing temperature of 205 °C. The number of layers of the welding sample is 398. The printing time of the welding sample is about 98 min. Figure 3 shows the FRW of the PLA-containing Al and PLA-containing Cu powders. The basic process of RFW is that one welding specimen is held stationary while the other is rotated at a constant rotation speed. To prevent one welding specimen under pressure from rotating with a rotating welding specimen, a fixture is designed to clamp the welding specimen on the stationary side. Two welding specimens are joined together under axial force. The whole welding process involves eight steps [26]: (a) setting two welding specimens, (b) applying force to the welding specimen on the stationary side, (c) rotating one welding specimen at the rotary side, (d) initial stage of the FRW, (e) middle stage of the FRW, (f) final stage of the FRW, (g) FRW is finished, and (h) the weld joint is processed. Based on many years of RFW experience [27], the whole welding time is 60 s. The cycle time of the RFW includes a friction time, welding time, and cooling time of 30 s, 20 s, and 10 s, respectively. The welding force, welding pressure, burn-off length, feed rate, and rotational speed are about 17 N, 0.054 MPa, 2 mm, 0.1 mm/s, and 950 rpm, respectively. A load cell (ARI742, Zhiheng Industrial Co., Inc., New Taipei City, Taiwan) was employed to measure welding force during RFW. The peak temperature of the weld joint during FRW was measured using an infrared camera (BI-TM-F01P, Panrico trading Inc., New Taipei City, Taiwan). The microstructure of the welded joint was investigated using field-emission scanning electron microscopy (FE-SEM) (JEC3000-FC, JEOL Inc., Tokyo, Japan). The macrostructure of the welded joint was investigated using optical microscopy (OM) (Quick Vision 404, Mitutoyo Inc., Tokyo, Japan). The bending strength of the welded parts was investigated using a three-point bending test machine (RH-30, Shimadzu Inc., Kyoto, Japan) with a movement speed of about 1 mm/s [28]. Figure 4 shows the experimental setup for the surface hardness and bending strength of the frictionally welded parts. The welded specimens were cut in half to have a flat surface for measurement. Figure 5 shows a schematic diagram of the surface hardness measurement location. The measurement points for the hardness distributions of the welded parts include twenty points. The measurement points of the welded joint include ten points. The thermal properties of the welded joint were investigated using differential scanning calorimetry (DSC) (STA 409 PC Luxx Simultaneous thermal analyzer, Netzsch-Gerätebau GmbH Inc., Hanau, Germany). The glass transition temperature (T_g_), melting point (T_m_), crystallization temperature (T_c_), and thermal decomposition (T_d_) can be determined after the DSC experiment. About 15 mg of the welded joint sample was put in platinum crucibles. The specimens were heated at a temperature ranging from 20 °C to 900 °C under a nitrogen gas flow rate of about 25 cc/min. A heating rate and a cooling rate of about 10 °C/min were employed during the DSC experiment. In addition, the welded parts were placed in ten different ambient temperatures to investigate the bending strength of the welded parts under different ambient temperatures. The ten different ambient temperatures included 25, 27, 30, 32, 34, 36, 38, 40, 43, and 45 °C, respectively.

## 3. Results and Discussion

**Figure 6:**
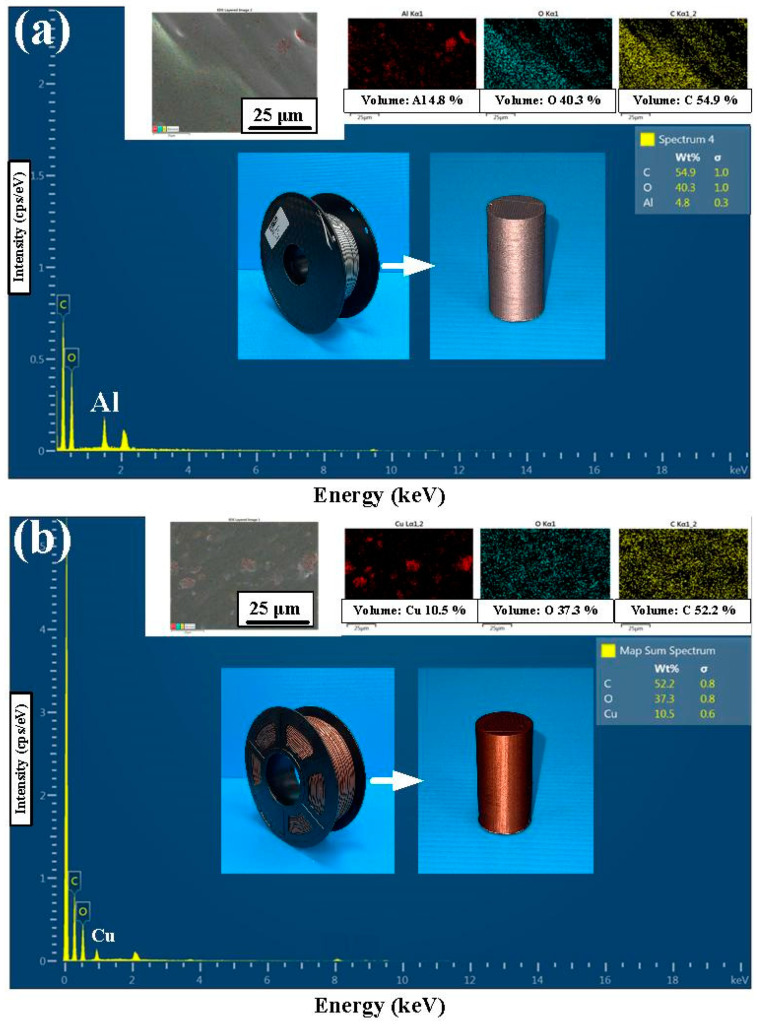
The chemical composition of the filaments of (**a**) PLA-containing Al and (**b**) PLA-containing Cu powders for making the welding specimens.

**Figure 7:**
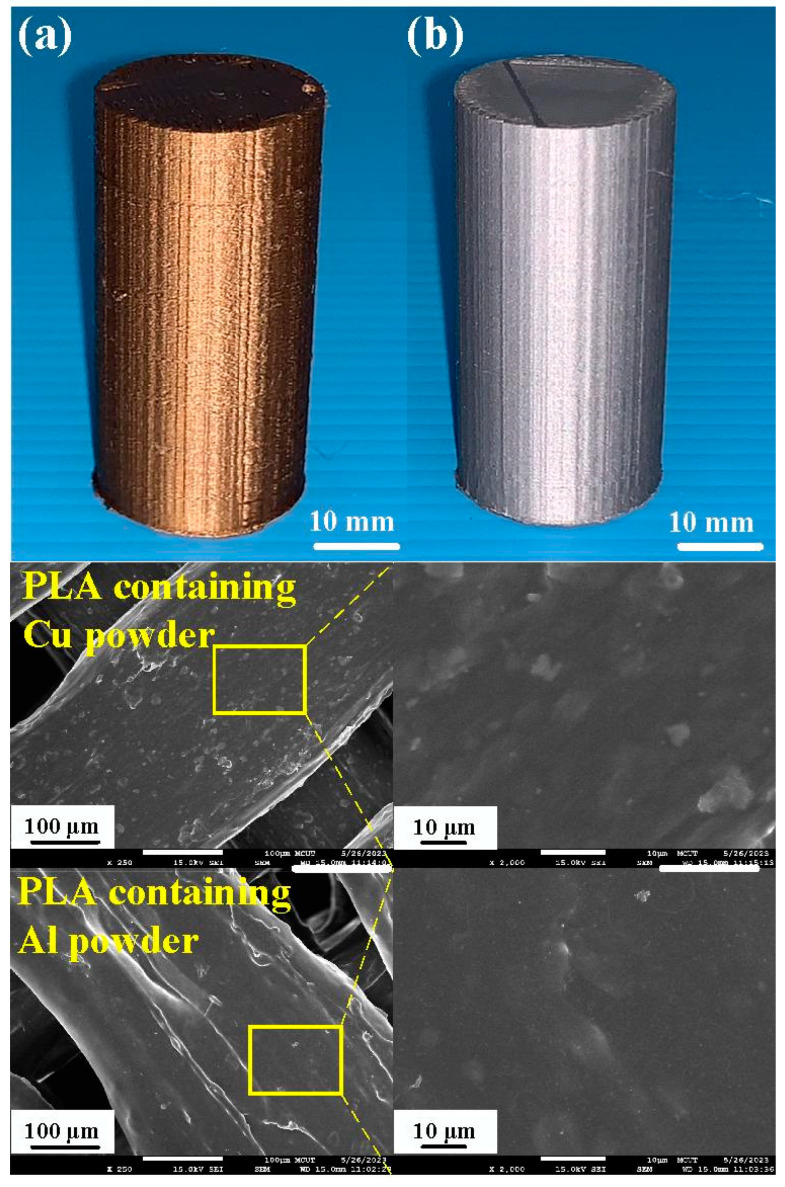
FE-SEM micrograph of two different RFW samples: (**a**) PLA-containing Cu and (**b**) PLA-containing Al powders.

Figure 6 shows the chemical composition of the filaments of PLA-containing Al and Cu powders for making the welding specimens. The chemical compositions of the two polymer filaments were characterized using energy-dispersive X-ray spectroscopy (JEC3000-FC, JEOL Inc., Tokyo, Japan). As can be seen, the content of the Cu and Al components in the PLA filament is about 10.5 wt.% and 4.8 wt.%, respectively. Figure 7 shows the FE-SEM micrograph of two different RFW samples for PLA-containing Cu and PLA-containing Al powders. The results showed that the gray area is the PLA material, and the white area represents the metal filler, i.e., the Al or Cu element. Significantly, it was found that the Al element was distributed in the PLA filament uniformly. However, the Cu element was not uniformly distributed in the PLA filament because of agglomeration [29,30], showing that part of the Cu powder is agglomerated.

**Figure 8:**
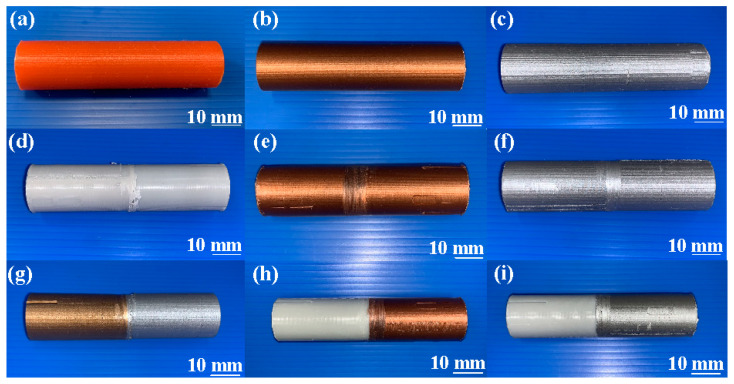
Nine different kinds of specimens for evaluating mechanical properties: (**a**) pure PLA, (**b**) PLA-containing Cu powder, (**c**) PLA-containing Al powder, (**d**) RFW of PLA and PLA, (**e**) RFW of PLA-containing Cu powder and PLA-containing Cu powder, (**f**) RFW of PLA-containing Al powder and PLA-containing Al powder, (**g**) RFW of PLA-containing Al powder and PLA-containing Cu powder, (**h**) RFW of PLA and PLA-containing Cu powder, and (**i**) RFW of PLA and PLA-containing Al powder.

In this study, five test specimens were used to perform RFW. Figure 8 shows the nine different kinds of specimens for evaluating mechanical properties. These specimens were made from pure PLA filament, PLA-containing Cu powder filament, PLA-containing Al powder filament, RFW of a PLA rod and a PLA rod, RFW of a PLA-containing Cu powder rod and a PLA-containing Cu powder rod, RFW of a PLA-containing Al powder rod and a PLA-containing Al powder rod, RFW of a PLA-containing Al powder rod and a PLA-containing Cu powder rod, RFW of PLA and PLA-containing Cu powder, as well as RFW of PLA and PLA-containing Al powder.

**Figure 9:**
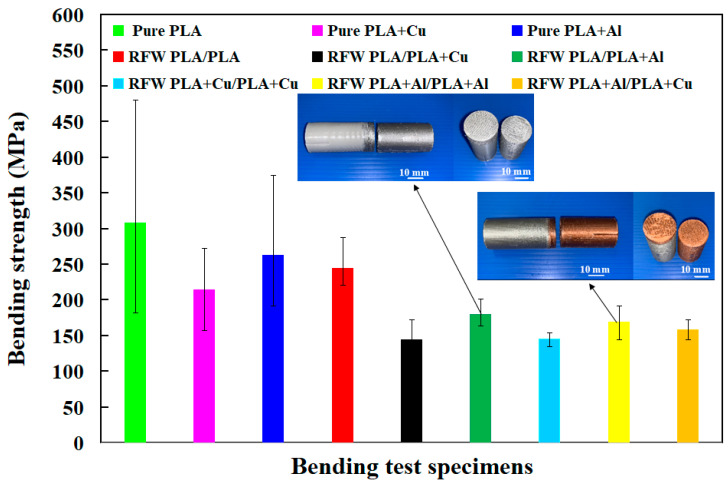
Bending strength of RFW specimens in comparison with pure materials.

**Figure 10:**
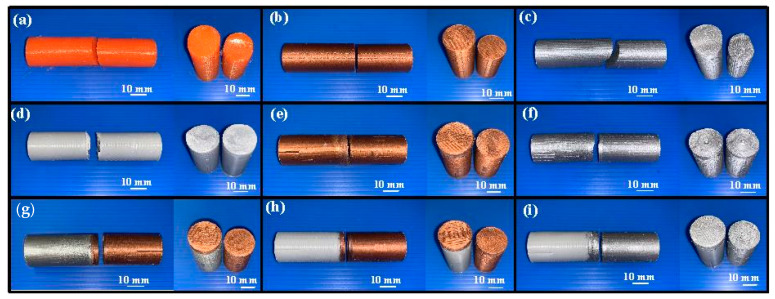
Bending test results of nine different specimens: (**a**) pure PLA, (**b**) PLA-containing Cu powder, (**c**) PLA-containing Al powder, (**d**) RFW of PLA and PLA, (**e**) RFW of PLA-containing Cu powder and PLA-containing Cu powder, (**f**) RFW of PLA-containing Al powder and PLA-containing Al powder, (**g**) RFW of PLA-containing Al powder and PLA-containing Cu powder, (**h**) RFW of PLA and PLA-containing Cu powder, and (**i**) RFW of PLA and PLA-containing Al powder.

In this study, five test specimens were used to perform RFW. The bending strength of the RFW specimens and the pure materials are summarized in Figure 9. Figure 10 shows the bending test results of the nine different specimens. Four phenomena were found: (a) The bending strength of the integrally formed bending test specimen is better than that of the bending test specimen fabricated by RFW. The average bending strength of the bending test specimen formed integrally with PLA-containing Cu powder is about 214.8 MPa. However, the average bending strength of the bending test specimen fabricated by RFW is only about 145.2 MPa. The reduction in bending strength is about 32%. This phenomenon is consistent with the research results of Mehri et al. [31]. (b) The bending strength of the bending test specimen integrally formed with PLA-containing Cu powder or PLA-containing Al powder is lower than that of the pure PLA integrally formed bending test specimen. A possible reason is that the distribution of Cu powder or Al powder in the PLA material is uneven or the content of Cu powder or Al powder in the PLA material is too low. Additionally, Cu powder or Al powder has poor compatibility [32,33] with PLA matrix materials, which leads to cracks or peeling of the bending test specimen during the bending test. (c) The average bending strength of the bending test specimen fabricated by RFW is higher than that of the cylinder printed with PLA-containing Cu powder. The bending strength was increased by about 16%. (d) The average bending strength of the bending test specimen is about 158.4 MPa for RFW of dissimilar polymer rods printed with PLA-containing Cu powder and PLA-containing Al powder, which is between the RFW of polymer rods printed with PLA-containing Al powder and the RFW of polymer rods printed with PLA-containing Cu powder.

**Figure 11:**
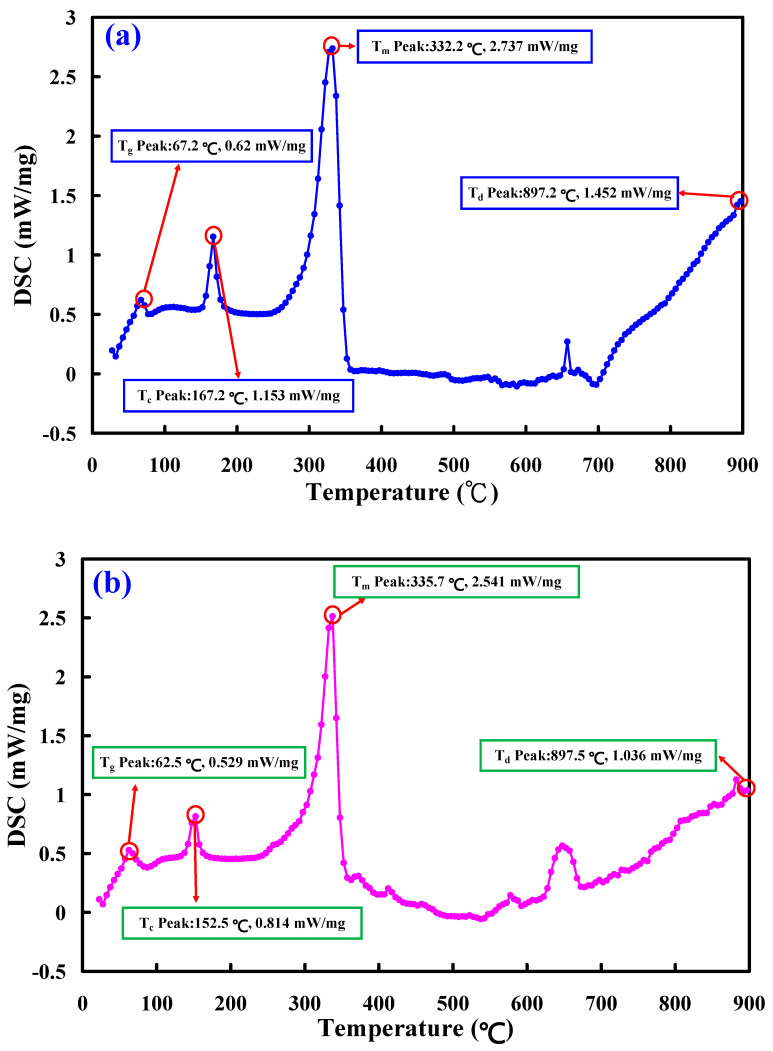
Thermal analyses of the two welded parts: (**a**) RFW of PLA-containing Al powder and PLA-containing Al powder rod and (**b**) RFW of PLA-containing Cu powder and PLA-containing Cu powder rod.

Figure 11 shows the thermal analyses of the two welded parts. As can be seen, the heat capacity [34] for RFW of PLA-containing Al powder and the PLA-containing Al powder rod and RFW of PLA-containing Cu powder and the PLA-containing Cu powder rod is about 2.737 mW/mg and 2.541 mW/mg. This means that the welded part fabricated by RFW of the PLA-containing Al powder and the PLA-containing Al powder has a higher molecular orientation in the welded joints than that of the welded part fabricated by RFW of the PLA-containing Cu powder and the PLA-containing Cu powder [35,36].

**Figure 12:**
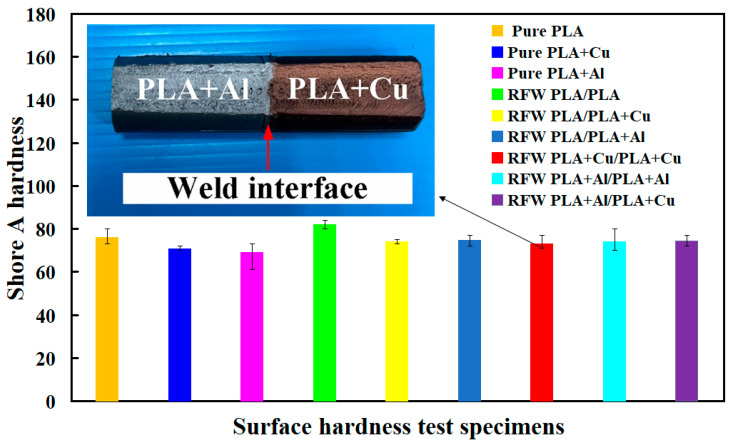
Surface hardness of RFW specimens in comparison to pure materials.

Figure 12 shows the surface hardness of RFW specimens in comparison to pure materials. The average surface hardness of the 3D-printed parts of three base materials and the 3D-printed parts of six different materials is about Shore A 76, 70.7, 69, 82.1, 74.1, 74.7, 73, 74, and 74.5, respectively. Two phenomena were found. One is that the surface hardness of the weld interface is better than that of the 3D-printed parts. The average surface hardness of the weld interface by RFW of the PLA-containing Al powder and the PLA-containing Al powder rods is about Shore A 74. However, the average surface hardness of the 3D-printed parts using PLA filament containing Al powder is only about Shore A 69. The surface hardness is increased by about 7.24%. The average surface hardness of the weld interface by RFW of the PLA-containing Cu powder and the PLA-containing Cu powder rods is about Shore A 70.7. However, the average surface hardness of the 3D-printed parts using PLA filament containing Cu powder is only about Shore A 73. The surface hardness is increased by about 3.25%. The average surface hardness of the weld interface by RFW of PLA and PLA rods is about Shore A 82.1. However, the average surface hardness of the 3D-printed parts using PLA filament is only about Shore A 76. The surface hardness is increased by about 8.02%. The other finding is that the average surface hardness of the weld interface from RFW of PLA and PLA is the highest.

**Figure 13:**
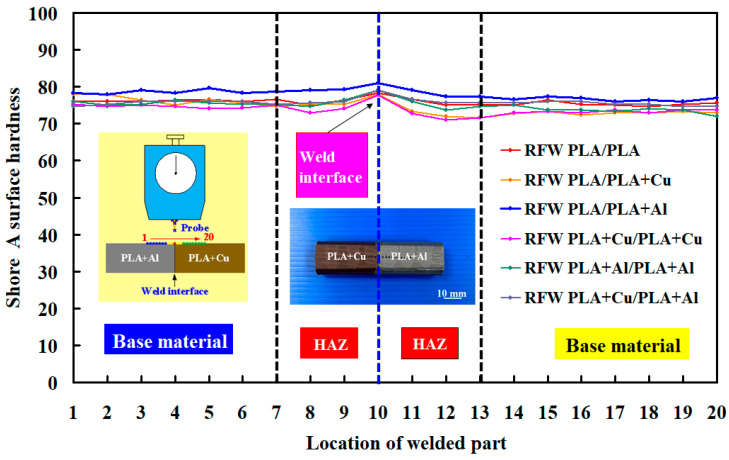
Surface hardness distributions of the welded part.

Figure 13 shows the surface hardness distributions of the welded part. According to the above results, two phenomena are found. One is that the surface hardness of the welded joint is higher than that of the base materials. The surface hardness increase rate is about 3%. The other finding is that the surface hardness of the heat-affected zone (HAZ) is lower than that of both the base materials and welded joint. The surface hardness reduction rate is about 3%. It should be noted that the HAZ for RFW of polymers is narrow due to the short time heating and cooling compared with RFW of metals.

**Figure 14:**
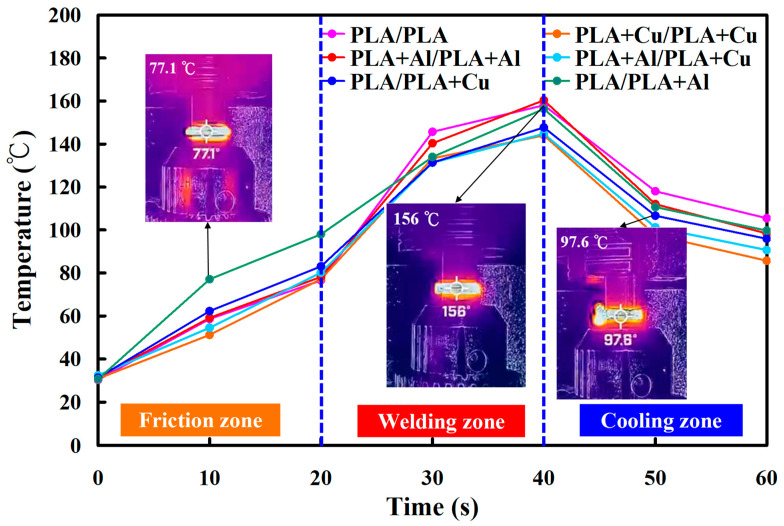
Temperature as a function of time in the weld interface for six welded parts.

**Figure 15:**
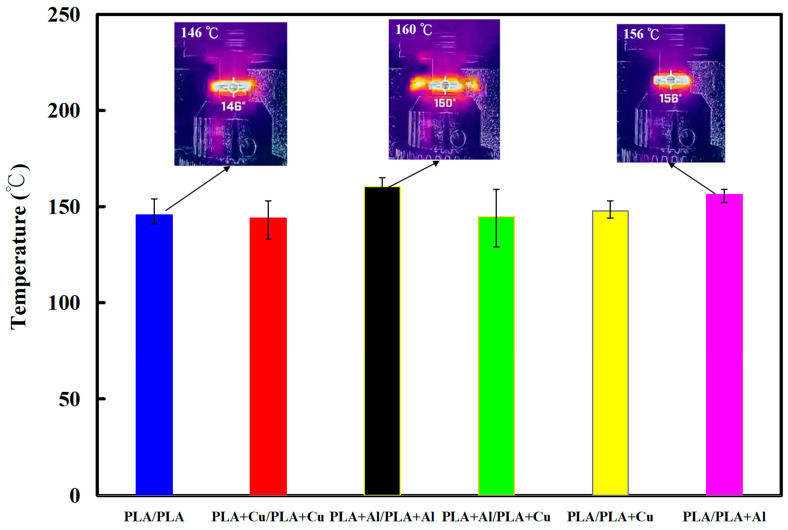
Peak temperatures in the weld interface for six welded parts.

Figure 14 shows the temperature as a function of time in the weld interface for six welded parts. Figure 15 shows the peak temperatures in the weld interface for six welded parts. The results revealed that the average peak temperatures of the six RFW processes are about 146 °C, 144 °C, 160 °C, 145 °C, 148 °C, and 156 °C. Two phenomena were found according to these results. The average peak temperature of the welded joint is the highest in the RFW of PLA-containing Al powder and PLA-containing Al powder rods. The average peak temperature of the welded joint can be as high as 160 °C. However, the average peak temperature of the welded joint is the highest in the RFW of PLA-containing Cu powder and PLA-containing Cu powder rods. The average peak temperature of the welded joint can be as high as 144 °C.

**Figure 16:**
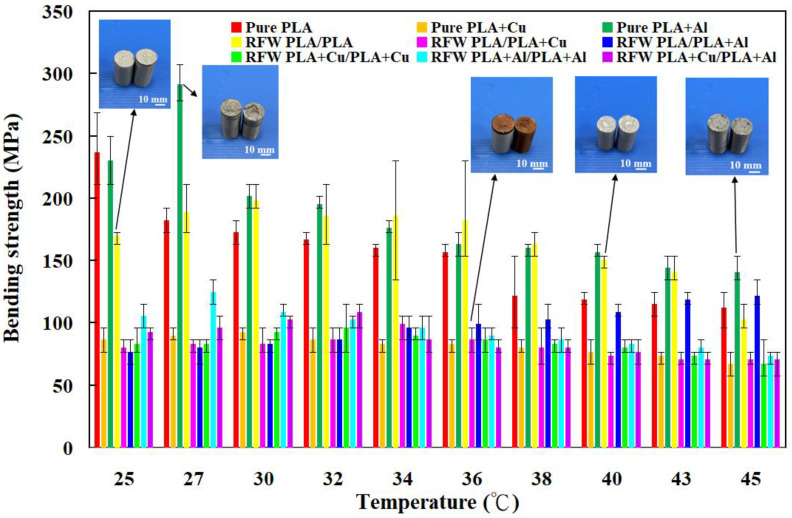
Bending strength of the welded parts under different ambient temperatures.

To investigate the bending strength of the welded parts under different ambient temperatures, the welded parts were put in ten different ambient temperatures, i.e., 25, 27, 30, 32, 34, 36, 38, 40, 43, and 45 °C. Figure 16 shows the bending strength of the welded parts under different ambient temperatures. The bending strength cannot be measured due to the test specimen becoming soft when the test specimen was placed above 45 °C. Two phenomena were observed. One is that the bending strength of the welded parts was affected by the ambient temperature greatly. This result shows that the mechanical property of the welded parts is also related to the ambient temperature used for welded components. The other finding is that the bending strength of the welded parts fabricated by RFW of PLA and PLA-containing Al powder rods could be enhanced by about 57.5% when the welded part was placed at 45 °C. Thus, this database can be used for the selection of ambient temperatures for the three base materials and six welded parts with dissimilar materials.

In practice, large physical models must be printed in separate pieces. Bolts play a crucial role in the industry. They are widely utilized to join small 3D-printed components to construct substantial 3D physical models. Nevertheless, these sizable physical models lack resilience against vibrations when constructed with this method, particularly when incorporating mobile components into their design. The quality of the frictionally welded parts relies on various welding process parameters within the realm of RFW. A welded part fabricated by PLA-containing copper powder and PLA-containing aluminum powder using RFW provides some advantages, such as enhancement of the thermal and electrical conductivity. Thus, there are several potential applications, such as heat sinks, circuit boards, electromagnetic shielding, electronic devices, heat exchangers, or aerospace applications. The sustainable development goals (SDGs) [37,38] are seventeen global goals established by the United Nations. These goals were created to address a wide range of economic, social, and environmental challenges facing the world and to promote a more sustainable and equitable future for all. Significantly, RFW is a green manufacturing process [39] compared with arc welding or ultrasonic welding [40,41,42,43]. This is consistent with sustainable development goal 12 [44,45,46]. The major disadvantages of ultrasonic welding are that it is limited to welding smaller or thinner plastic parts. It should be noted that RFW is suitable for welding thicker or large components. Unfortunately, the crack growth rate was not investigated using the Paris law [47]. Thermogravimetric analysis [48] is also an alternative for thermal analysis of welded parts after RFW. Fatigue testing is recommended to assess the quality of welded parts fabricated by PLA-containing copper powder and PLA-containing aluminum powder [49]. In this study, RFW is employed to join polymer rods fabricated by PLA-containing copper powder and PLA-containing aluminum powder. Friction stir welding is also recommended for jointing polymer rods fabricated with PLA-containing copper powder and PLA-containing aluminum powder [50,51]. This topic is an exciting research topic and is currently being investigated.

## 4. Conclusions

Additive manufacturing with polymers is widely used for manufacturing a variety of functional plastic components. However, the additive manufacturing machine has a substantial limitation in the size of functional components. RFW is a possible solution to this obstacle. Two welding specimens fabricated with polylatic PLA-containing copper powder and PLA-containing aluminum powder were joined by RFW. After RFW, welding quality was evaluated by Shore A surface hardness tests and a bending test. The main conclusions from the experimental work in this study are as follows:

1. A technical database serves as a valuable resource for determining the appropriate ambient temperatures for three base materials and six welded parts with dissimilar materials. The mechanical characteristics of the welded components are influenced by the ambient temperature during the welding process. Notably, the bending strength of welded parts produced by RFW using PLA and PLA-containing Al powder rods can be increased by approximately 57.5% when the welding occurs at a temperature of 45 °C.
2. The surface hardness of the weld interface surpasses that of the 3D-printed components. Notably, the average surface hardness of the weld interface resulting from the RFW of PLA to PLA is the highest among the tested cases.
3. The average peak temperature of the welded joint is the highest in the RFW of PLA-containing Al powder and PLA-containing Al powder rods. The average peak temperature of the welded joint can be as high as 160 °C. However, the average peak temperature of the welded joint is the highest in the RFW of PLA-containing Cu powder and PLA-containing Cu powder rods. The average peak temperature of the welded joint can be as high as 144 °C.
4. The heat capacity for RFW of PLA-containing Al powder and PLA-containing Al powder rods and RFW of PLA-containing Cu powder and PLA-containing Cu powder rods is about 2.737 mW/mg and 2.541 mW/mg, showing the welded part fabricated by RFW of PLA-containing Al powder and PLA-containing Al powder has better weld strength.

## Data Availability

Data and materials are available.
